# (5R)-5-Hydroxytriptolide (LLDT-8) Ameliorates Experimental Autoimmune Myositis via Suppression of the NLRC5/MHC-I Signaling Pathway

**DOI:** 10.3390/ph19040631

**Published:** 2026-04-17

**Authors:** Tingting Hao, Qing Qi, Cancan Xie, Li Chen, Meijuan Shao, Que Wang, Zemin Lin, Fenghua Zhu, Xiaoqian Yang, Shijun He, Jianping Zuo

**Affiliations:** 1 Experiment Center for Science and Technology, Shanghai University of Traditional Chinese Medicine, Shanghai 201203, China; haoting0628@163.com (T.H.); xcc51217@163.com (C.X.); shaomeijuan@jxutcm.edu.cn (M.S.); 18616833881@163.com (Q.W.); 2 State Key Laboratory of Drug Research, Shanghai Institute of Materia Medica, Chinese Academy of Sciences, Shanghai 201203, China; 201728012342034@simm.ac.cn (L.C.); linzemin@simm.ac.cn (Z.L.); fhzhu@simm.ac.cn (F.Z.); xqyang@simm.ac.cn (X.Y.); 3 School of International Equestrianism, Wuhan Business University, Wuhan 430056, China; 4 Innovation Research Institute of Traditional Chinese Medicine, Shanghai University of Traditional Chinese Medicine, Shanghai 201203, China; heshijun@shutcm.edu.cn

**Keywords:** C2C12 myoblasts, antigen presentation, experimental autoimmune myositis, triptolide derivative, molecular docking, muscle weakness

## Abstract

**Background:** Idiopathic inflammatory myopathies (IIMs), characterized by muscle weakness and chronic inflammation, currently lack highly effective therapies. This study investigated the therapeutic potential and underlying mechanism of (5R)-5-hydroxytriptolide (LLDT-8), a triptolide derivative with reduced toxicity, using an experimental autoimmune myositis (EAM) mouse model and in vitro assays. **Methods:** Forty female BALB/c mice were randomly assigned to five groups: normal, vehicle, methylprednisolone (MP), LLDT-8 (0.0625 mg/kg), and LLDT-8 (0.125 mg/kg). EAM mice were treated with LLDT-8 (0.0625 or 0.125 mg/kg) or methylprednisolone as a positive control. Cellular experiments and molecular docking were performed to investigate potential mechanisms of LLDT-8. **Results:** LLDT-8 significantly attenuated clinicopathological features, including muscle weakness and pain sensitivity, while reducing serum levels of aspartate aminotransferase and lactate dehydrogenase. Histological analysis revealed that LLDT-8 reduced inflammatory cell infiltration and the presence of CD4^+^ and CD8^+^ T cells in muscle tissues. Mechanistically, LLDT-8 inhibited the expression of nucleotide-binding oligomerization domain receptor caspase recruitment domain 5 (NLRC5), a key transcriptional regulator of major histocompatibility complex-I (MHC-I). This suppression extended to downstream antigen presentation-related molecules, including the transporter associated with antigen processing and proteasome 20S subunit beta. Molecular docking further confirmed the high binding affinity of LLDT-8 to both NLRC5 and MHC-I. **Conclusions:** LLDT-8 alleviates inflammatory muscle injury by targeting the NLRC5/MHC-I signaling axis, suggesting it may be a promising therapeutic candidate for IIMs.

## 1. Introduction

Idiopathic inflammatory myopathies (IIMs) are a heterogeneous group of autoimmune disorders characterized by progressive muscle weakness, chronic muscle inflammation, and elevated serum muscle enzymes [1,2]. The incidence of IIMs ranges from 0.2 to 2 cases per 100,000 person-years and varies by sex, with a higher prevalence observed in females, particularly those with dermatomyositis [3]. Despite advances in diagnosis and management, up to 40% of patients with IIMs die within five years of diagnosis [3], imposing substantial burdens on patients, their families, and healthcare systems [4,5]. The development of more effective therapeutic strategies therefore remains an urgent clinical need.

IIMs are usually classified into four subtypes based on the clinicopathologic features: dermatomyositis, polymyositis, necrotizing auto-immunology and inclusion-body myositis [1]. Polymyositis and dermatomyositis, two largely distinct subgroups of IIMs, are characterized by muscle weakness and inflammatory infiltration in muscle [6]. Polymyositis mainly affects the skeletal muscles and is accompanied by abundant infiltration of CD8^+^ T lymphocytes, suggesting an immune-mediated mechanism in polymyositis [1,6,7]. Dermatomyositis mainly affects the skin and muscles; for instance, CD4^+^ T cells, plasmacytoid dendritic cells, and B lymphocytes were found to be more common in muscle tissue from patients with dermatomyositis [3,8].

Widespread overexpression of major histocompatibility complex class I (MHC-I) on the surface of muscle fibers is considered a hallmark of IIM and may precede overt inflammatory infiltration [9,10]. MHC-I molecules have a significant impact on the development and function of the immune system, with their classic function being to transport endogenous antigen peptides to the cell membrane surface and present the peptide antigens to CD8^+^ T cells through TCR, thereby activating cytotoxicity [11]. In one study, the upregulation of MHC-I in skeletal muscle fibers was found in the early stage of inflammatory cell infiltration in polymyositis. Therefore, we believe that the abnormal upregulation of MHC-I in muscle fibers may be the initiating factor of muscle damage [12].

Further studies have shown that immature muscle fiber precursors and regenerating fibers are important sources of polymyositis autoantigens, and the overexpression of MHC-I mainly occurs on the microsurface of immature and regenerating muscle fibers [13,14]. Moreover, aberrant MHC-I expression promotes the release of muscle-derived cytokines and chemokines, thereby enhancing T-cell chemotaxis [15]. For example, CD8^+^ T cells have been observed surrounding MHC-I–expressing muscle fibers [16], and, in a study of transgenic myopathic mice, sustained MHC-I overexpression impairs muscle force generation [17]. Conversely, proinflammatory cytokines can further induce MHC-I expression in muscle cells [15,18], highlighting a pathogenic amplification loop that contributes to muscle injury in IIM.

Nucleotide-binding oligomerization domain receptor caspase recruitment domain 5 (NLRC5) is a key transcriptional regulator of MHC-I and its associated antigen-processing machinery, including proteasome 20S subunit beta 8/9 (Psmb8/9), beta-2 microglobulin (β2M), and the transporter associated with antigen processing 1/2 (Tap1/2) [19,20]. Reduction of NLRC5 decreases MHC-I transcription and surface expression [21], and it has been found that NLRC5-deficient mice exhibit impaired CD8^+^ T-cell function due to diminished MHC-I expression on hematopoietic cells [22]. These findings suggest that dysregulation of the NLRC5/MHC-I axis may play a critical role in the pathogenesis of IIM.

Triptolide, an active compound extracted from the traditional Chinese medicinal herb *Tripterygium wilfordii* Hook F, has demonstrated potent pharmacological activity in multiple studies [23]. (5R)-5-Hydroxytriptolide (LLDT-8), one of its structurally modified derivatives, exhibits improved efficacy and reduced toxicity compared with its parent compound [24,25], and accumulating evidence indicates that LLDT-8 exerts therapeutic effects in various autoimmune and inflammatory disease models, including rheumatoid arthritis [26], dermatitis [27], non-alcoholic fatty liver disease [28], and certain malignancies [25]. However, its potential role in IIMs remains unclear.

As an experimental autoimmune myositis (EAM) model established by immunization with skeletal muscle globulin can successfully mimic the characteristics of human IIMs [8], in this study, we evaluated the therapeutic effects of LLDT-8 in a murine EAM model and a complementary in vitro system to address this research gap. Disease severity was assessed through measurements of muscle strength, serum biochemical parameters, and histopathological changes in muscle tissue. Furthermore, the underlying mechanisms were explored in interferon-γ (IFN-γ)-stimulated C2C12 myoblasts and differentiated myotubes, and molecular docking analysis was performed to predict potential target interactions.

## 2. Results

### 2.1. LLDT-8 Treatment Attenuated Clinical Scores, Visceral Indexes, and Biochemical Indicators in the EAM Mice

**Figure 1:**
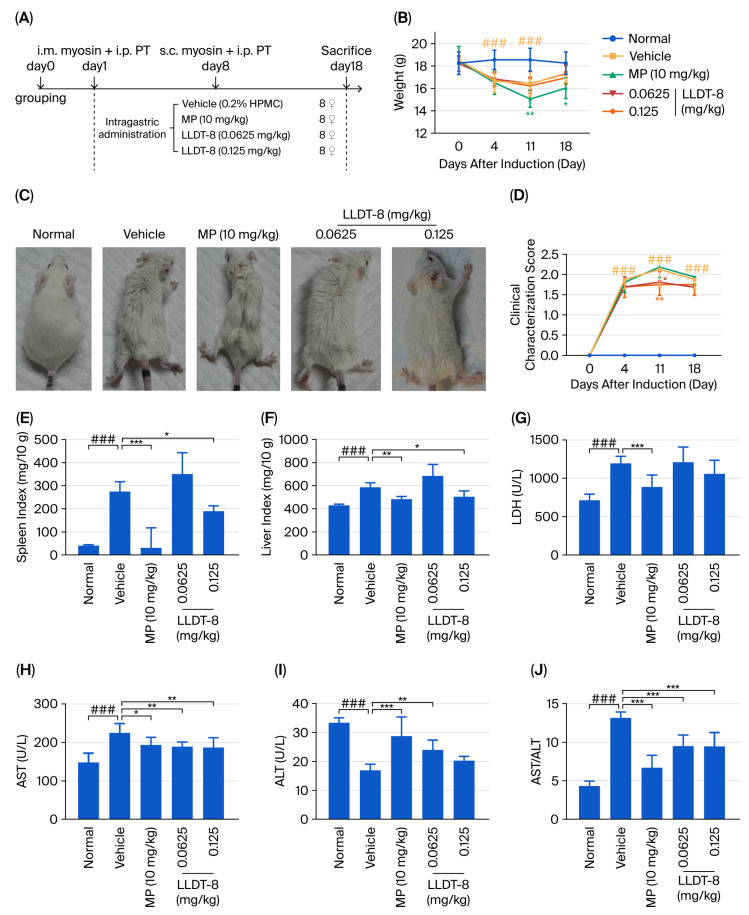
Effect of (5R)-5-hydroxytriptolide (LLDT-8) on EAM mice. (**A**) Experimental schedule of animal modeling and drug administration. (**B**) Body weight changes in each group at the indicated time points. (**C**) Representative phenotypic characteristics of mice in different groups. (**D**) Clinical scores at different time points. (**E**) Spleen and (**F**) liver indexes of each group. The organ index was calculated as organ wet weight/body weight (mg/10 g). (**G**–**J**) Serum levels of lactate dehydrogenase (LDH) (**G**), aspartate aminotransferase (AST) (**H**), and alanine aminotransferase (ALT) (**I**), and the AST/ALT ratio (**J**). *n* = 8. ^###^ *p* < 0.001 indicate vehicle group vs. normal group; * *p* < 0.05, ** *p* < 0.01, *** *p* < 0.001 indicate vehicle group vs. drug-treated groups.

EAM was established as a classical IIM animal model to evaluate the in vivo efficacy of LLDT-8 [29]. Methylprednisolone (MP) was used as a positive control [30]. Mice in the normal group exhibited normal behavior and steady weight gain throughout the study (Figure 1A–C), while mice in the vehicle group developed evident clinical manifestations, including rough and unkempt fur, reduced body weight and activity, apparent myasthenia, and occasional lateral recumbency (Figure 1B,C). Notably, the mean clinical score in the vehicle group exceeded 2 points by day 11 after induction, which was significantly higher than that in the normal group (Figure 1D). Although LLDT-8 did not significantly affect body weight, both doses improved general appearance and behavior (Figure 1B,C) and significantly reduced clinical scores (Figure 1D). In contrast, mice in the MP group exhibited marked weight loss following the second immunization (Figure 1B).

Visceral indexes and muscle-related biochemical parameters are commonly used to assess disease severity and therefore were utilized in this study. The vehicle group displayed splenomegaly (Figure 1E), accompanied by significantly increased spleen and liver indexes (Figure 1E,F). Treatment with LLDT-8 (0.125 mg/kg) or MP significantly reduced these indexes, whereas LLDT-8 (0.0625 mg/kg) showed no significant effect compared with that observed in the vehicle group (Figure 1E,F).

In the past, serum biochemical markers have been found to correlate with the severity of muscle inflammation and injury [31]. In this study, lactate dehydrogenase (LDH) levels were significantly elevated in the vehicle group (Figure 1G), MP showed a trend toward reducing LDH, and LLDT-8 had no significant effect (Figure 1G).

Lastly, we examined the aspartate aminotransferase (AST)/alanine aminotransferase (ALT) ratio, which is considered a potential indicator of disease exacerbation in IIM [32]. Compared with the normal group, the vehicle group exhibited increased AST levels (Figure 1H), decreased ALT levels (Figure 1I), and consequently an elevated AST/ALT ratio (Figure 1J). Both MP and LLDT-8 significantly reduced AST levels and the AST/ALT ratio while restoring ALT levels compared with the vehicle group (Figure 1H–J), suggesting an overall attenuation of disease severity.

### 2.2. LLDT-8 Relieved Myasthenia and Pain Sensitivity in the EAM Mice

**Figure 2:**
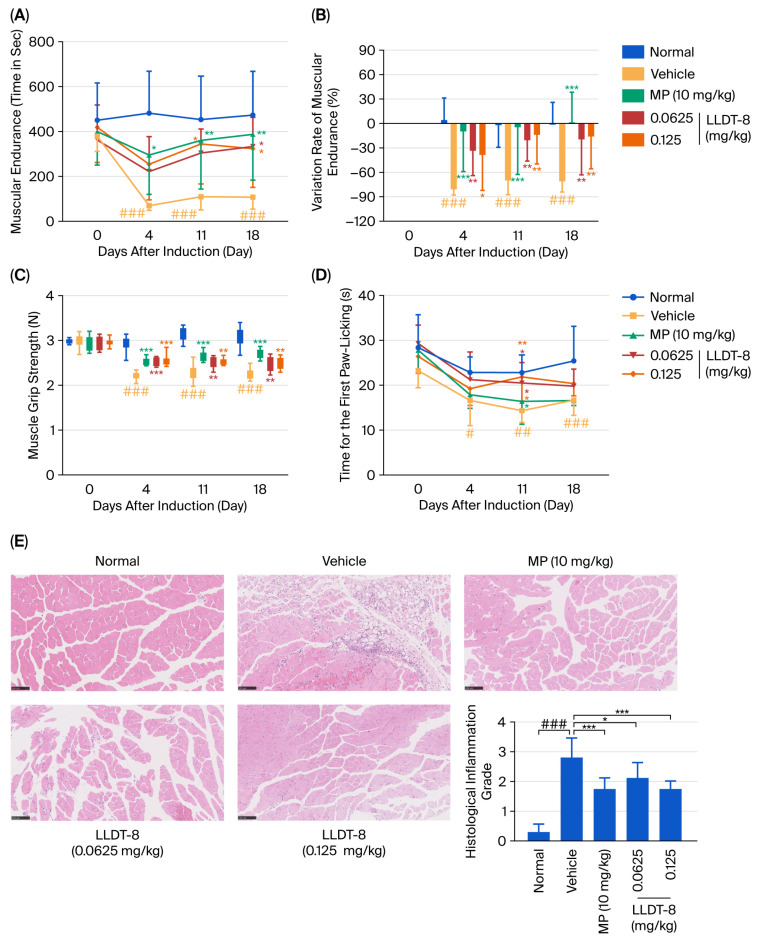
LLDT-8 improved muscle function and alleviated pathological changes in muscle tissue of EAM mice. (**A**) Muscular endurance in different groups. (**B**) Relative variation rate of muscular endurance, calculated as [(Day X − Day 0)/Day 0] × 100%. (**C**) Grip strength in different groups. (**D**) Pain sensitivity assessed by the hot plate test; the latency from placement on the hot plate to the first paw-licking response was defined as the pain threshold. (**E**) Representative H&E staining images and quantitative analysis of muscle tissues (scale bar = 100 μm). *n* = 8. ^#^ *p* < 0.05, ^##^ *p* < 0.01, ^###^ *p* < 0.001 indicate vehicle group vs. normal group; * *p* < 0.05, ** *p* < 0.01, *** *p* < 0.001 indicate vehicle group vs. drug-treated groups.

Myasthenia, heightened pain sensitivity, and inflammatory infiltration are key pathological features of IIM [33]. In this study, muscular endurance and grip strength were measured to assess muscle function. Compared with the normal group, mice in the vehicle group showed markedly reduced endurance (Figure 2A,B) and grip strength (Figure 2C), whereas treatment with MP or LLDT-8 (0.0625 and 0.125 mg/kg) significantly improved both metrics (Figure 2A–C). No significant differences were observed between the LLDT-8-treated and MP-treated groups. In the hot plate test, mice in the vehicle group exhibited a significantly shortened latency to the first paw-licking response from day 4 onward, indicating increased pain sensitivity (Figure 2D), while LLDT-8 treatment prolonged the paw-licking latency, with a significant improvement observed on day 11 (Figure 2D). These findings demonstrate that LLDT-8 effectively alleviated muscle weakness and hyperalgesia in EAM mice.

### 2.3. LLDT-8 Ameliorated Inflammatory Infiltration in the EAM Mice

Histopathological changes in muscle tissues were assessed by Hematoxylin and Eosin (H&E) staining. Normal mice exhibited intact muscle fibers with well-organized bundles and no evident inflammatory infiltration. In contrast, the vehicle group showed extensive inflammatory cell infiltration within muscle bundles, accompanied by fiber degeneration and necrosis, with a mean histological score of approximately 3 (Figure 2E). Treatment with MP or LLDT-8 (0.0625 and 0.125 mg/kg) markedly reduced inflammatory infiltration and alleviated muscle fiber damage (Figure 2E).

**Figure 3:**
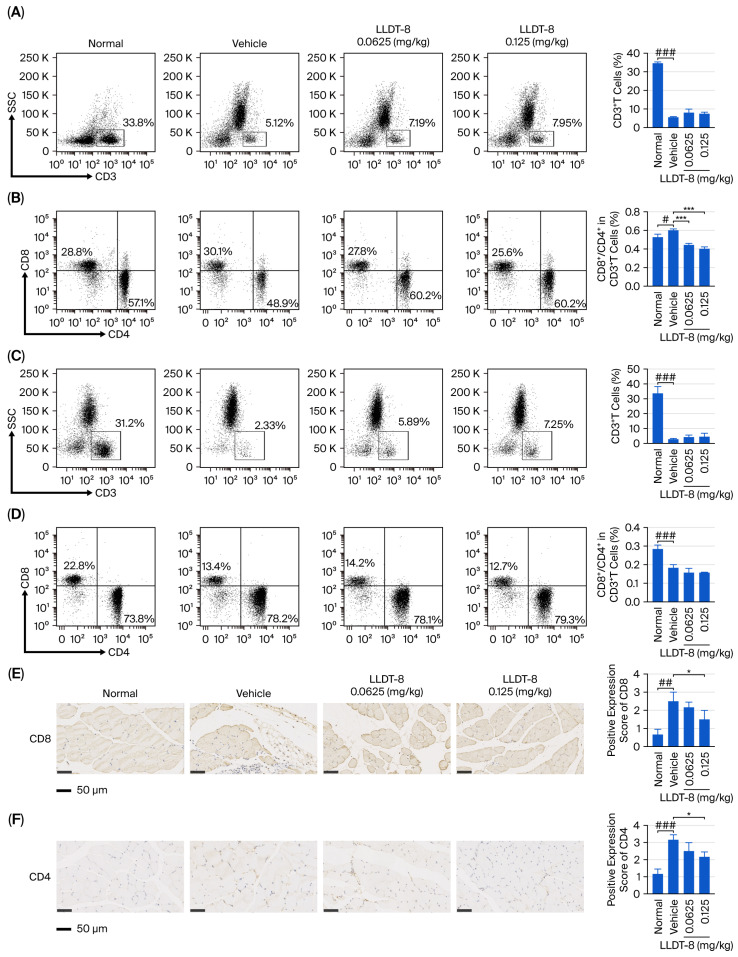
LLDT-8 attenuated the inflammatory response in EAM mice. The proportions of CD3^+^ T cells (**A**) and the CD8^+^/CD4^+^ ratio within gated CD3^+^ T cells (**B**) in murine splenocytes were analyzed by flow cytometry. The proportions of CD3^+^ T cells (**C**) and the CD8^+^/CD4^+^ ratio within gated CD3^+^ T cells (**D**) in murine peripheral blood mononuclear cells (PBMCs) were also evaluated. Representative immunohistochemical staining of CD8 (**E**) and CD4 (**F**) expression in muscle tissues from different groups is shown (scale bar = 50 μm). ^#^ *p* < 0.05, ^##^ *p* < 0.01, ^###^ *p* < 0.001; * *p* < 0.05, *** *p* < 0.001.

T cells are known to contribute to muscle injury in IIM [15,16]. Thus to further investigate the immunomodulatory effects of LLDT-8, we analyzed T-cell subsets in splenocytes and peripheral blood mononuclear cells (PBMCs). The vehicle group exhibited a reduced proportion of CD3^+^ T cells in both splenocytes (Figure 3A) and PBMCs (Figure 3C), while LLDT-8 treatment did not significantly alter the overall CD3^+^ T-cell proportion. Notably, the vehicle group showed an increased CD8^+^/CD4^+^ T-cell ratio in splenocytes (Figure 3B) and a decreased ratio in PBMCs (Figure 3D), while LLDT-8 treatment normalized the elevated CD8^+^/CD4^+^ ratio in splenocytes (Figure 3B) but had no significant effect on this ratio in PBMCs (Figure 3D).

Given that the reduced proportion of circulating CD8^+^ T cells might reflect their recruitment to inflamed muscle tissue, immunohistochemical staining was performed to evaluate CD8 and CD4 expression in muscle. The vehicle group showed marked infiltration of CD8^+^ and CD4^+^ T cells in muscle tissue (Figure 3E,F), while Treatment with LLDT-8 (0.125 mg/kg) significantly reduced the infiltration of both CD8^+^ and CD4^+^ T cells (Figure 3E,F), indicating attenuation of inflammatory damage in EAM mice.

### 2.4. LLDT-8 Suppressed the NLRC5/MHC-I Pathway in the EAM Mice

**Figure 4:**
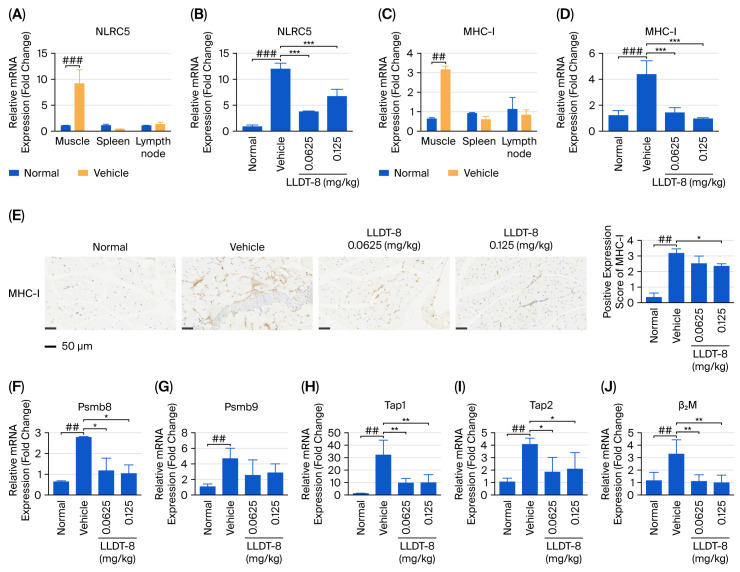
LLDT-8 suppressed activation of the NLRC5/MHC-I pathway in EAM mice. mRNA expression of *NLRC5* in muscle (**A**,**B**), spleen (**A**), and lymph nodes (**A**) and mRNA expression of *MHC-I* in muscle (**C**,**D**), spleen (**C**), and lymph nodes (**C**) were analyzed by qRT-PCR. (**E**) Representative immunohistochemical staining and semi-quantitative analysis of MHC-I expression in muscle tissue (scale bar = 50 μm). mRNA levels of *Psmb8* (**F**), *Psmb9* (**G**), *Tap1* (**H**), *Tap2* (**I**), and *β2M* (**J**) in muscle tissues from different groups are shown. ^##^ *p* < 0.01, ^###^ *p* < 0.001; * *p* < 0.05, ** *p* < 0.01, *** *p* < 0.001.

Aberrant NLRC5 expression can regulate MHC-I levels and modulate CD8^+^ T-cell responses [9,10,22] and processes critical in IIM-associated muscle injury [34]. Therefore to explore the molecular mechanism underlying the therapeutic effects of LLDT-8, we examined the expression of NLRC5 and its downstream target MHC-I in relevant tissues by qRT-PCR and immunohistochemistry. mRNA expression of *NLRC5* and *MHC-I* was assessed in muscle, spleen, and lymph nodes (Figure 4A,C), and, among these tissues, muscle displayed the highest expression levels of both *NLRC5* (Figure 4A) and *MHC-I* (Figure 4C) in EAM mice. LLDT-8 treatment significantly reduced the elevated expression of NLRC5 (Figure 4B) and MHC-I (Figure 4D), which was further confirmed by immunohistochemical analysis (Figure 4E). As a nuclear transcription factor, *NLRC5* regulates not only *MHC-I* but also key components of the antigen-processing machinery, including *β2M*, *Tap1/2*, and *Psmb8/9* [34]. Therefore, we evaluated the expression of these downstream genes in muscle tissue. The vehicle group showed significant upregulation of *Psmb8* (Figure 4F), *Psmb9* (Figure 4G), *Tap1* (Figure 4H), *Tap2* (Figure 4I), and *β2M* (Figure 4J), whereas treatment with LLDT-8 at both doses markedly reduced the transcriptional levels of these genes in vivo (Figure 4F–J), indicating suppression of the NLRC5/MHC-I axis in EAM mice.

### 2.5. LLDT-8 Suppressed the NLRC5/MHC-I Pathway in IFN-γ-Treated C2C12 Myoblasts In Vitro

**Figure 5:**
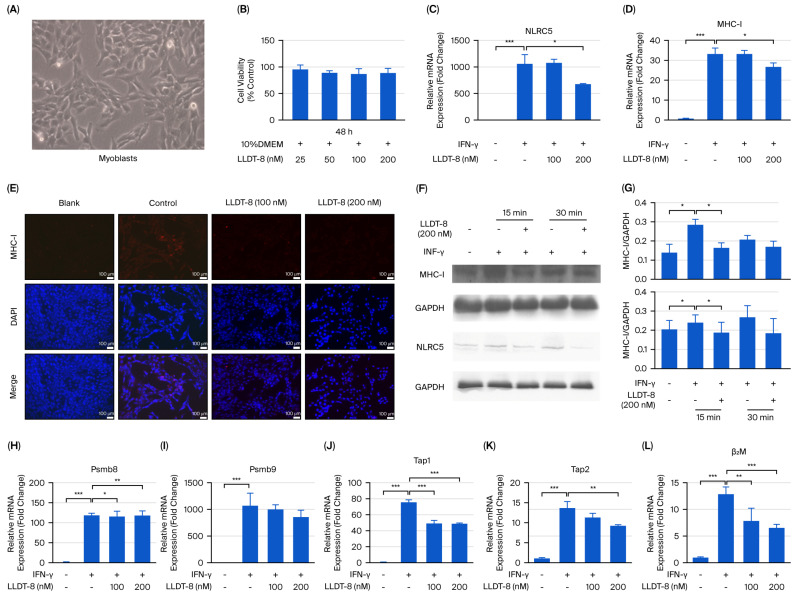
LLDT-8 inhibited activation of the NLRC5/MHC-I pathway in IFN-γ-treated C2C12 myoblasts in vitro. (**A**) Representative morphology of C2C12 myoblasts (scale bar = 200 μm). (**B**) Cell viability assessed by MTT assay. (**C**–**L**) C2C12 myoblasts were treated with or without IFN-γ and LLDT-8 (100 nM or 200 nM). mRNA expression of *NLRC5* (**C**) and *MHC-I* (**D**) was analyzed by qRT-PCR. (**E**) MHC-I expression was detected by immunofluorescence staining (scale bar = 100 μm). Protein levels of NLRC5 and MHC-I were analyzed by Western blotting (**F**,**G**), with GAPDH as an internal control. mRNA expression of *Psmb8* (**H**), *Psmb9* (**I**), *Tap1* (**J**), *Tap2* (**K**), and *β2M* (**L**) was determined by qRT-PCR. Experiments were repeated three times. * *p* < 0.05, ** *p* < 0.01, *** *p* < 0.001.

NLRC5 is a key regulator of MHC-I expression in interferon-mediated immune responses [35]. To determine whether LLDT-8 modulates the NLRC5/MHC-I pathway in vitro, we examined its effects in IFN-γ-treated C2C12 myoblasts (Figure 5), and based on the cell viability results (Figure 5A,B), two concentrations of LLDT-8 (100 nM and 200 nM) were selected for further investigation. IFN-γ stimulation significantly increased both mRNA and protein levels of NLRC5 and MHC-I, as demonstrated by qRT-PCR (Figure 5C,D), immunofluorescence (Figure 5E), and Western blotting (Figure 5F,G). Treatment with 200 nM LLDT-8, but not treatment with 100 nM, significantly reduced the IFN-γ-induced upregulation of *NLRC5* (Figure 5C) and *MHC-I* (Figure 5D) at the mRNA level. Consistently, 200 nM LLDT-8 markedly suppressed the elevated protein expression of NLRC5 and MHC-I (Figure 5E–G). We further examined the transcriptional levels of downstream antigen-processing genes, and it was found that IFN-γ treatment significantly upregulated *Psmb8* (Figure 5H), *Psmb9* (Figure 5I), *Tap1* (Figure 5J), *Tap2* (Figure 5K), and *β2M* (Figure 5L). In line with the in vivo findings (Figure 4), LLDT-8 significantly attenuated the IFN-γ-induced transcriptional upregulation of these genes in C2C12 myoblasts (Figure 5H–L), indicating effective suppression of the NLRC5/MHC-I axis in vitro.

### 2.6. LLDT-8 Suppressed the NLRC5/MHC-I Pathway in IFN-γ-Treated Myotubes In Vitro

**Figure 6:**
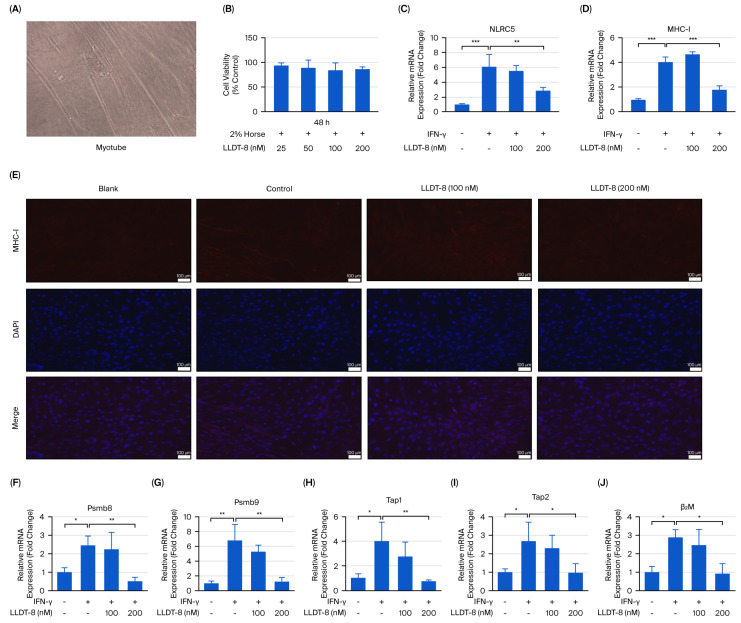
LLDT-8 suppressed activation of the NLRC5/MHC-I pathway in IFN-γ-treated myotubes in vitro. (**A**) Representative morphology of differentiated myotubes (scale bar = 200 μm). (**B**) Cell viability assessed by MTT assay. (**C**–**J**) Myotubes were treated with or without IFN-γ and LLDT-8 (100 nM or 200 nM). mRNA expression of *NLRC5* (**C**) and *MHC-I* (**D**) was measured by qRT-PCR. (**E**) MHC-I expression was examined by immunofluorescence staining (scale bar = 100 μm). mRNA levels of *Psmb8* (**F**), *Psmb9* (**G**), *Tap1* (**H**), *Tap2* (**I**), and *β2M* (**J**) were analyzed by qRT-PCR. Experiments were repeated three times. * *p* < 0.05, ** *p* < 0.01, *** *p* < 0.001.

To further validate these findings in differentiated muscle cells, we assessed the NLRC5/MHC-I pathway in IFN-γ-treated myotubes (Figure 6), and, based on the viability results (Figure 6A,B), LLDT-8 at 100 nM and 200 nM was selected for mechanistic evaluation. IFN-γ stimulation significantly increased *NLRC5* and *MHC-I* mRNA levels (Figure 6C,D), accompanied by elevated MHC-I protein expression (Figure 6E), while treatment with 200 nM LLDT-8 effectively reduced the IFN-γ-induced upregulation of NLRC5 and MHC-I at mRNA or protein levels (Figure 6C–E). Similarly, IFN-γ markedly increased the expression of downstream antigen-processing genes, including *Psmb8* (Figure 6F), *Psmb9* (Figure 6G), *Tap1* (Figure 6H), *Tap2* (Figure 6I), and *β2M* (Figure 6J), while LLDT-8 significantly attenuated the transcriptional upregulation of these genes in myotubes (Figure 6F–J). These results demonstrate that LLDT-8 suppresses IFN-γ-induced activation of the NLRC5/MHC-I pathway in both myoblasts and myotubes, suggesting that modulation of antigen presentation machinery may underlie its protective effects in EAM.

### 2.7. Molecular Docking of NLRC5 and MHC-I with LLDT-8

**Figure 7:**
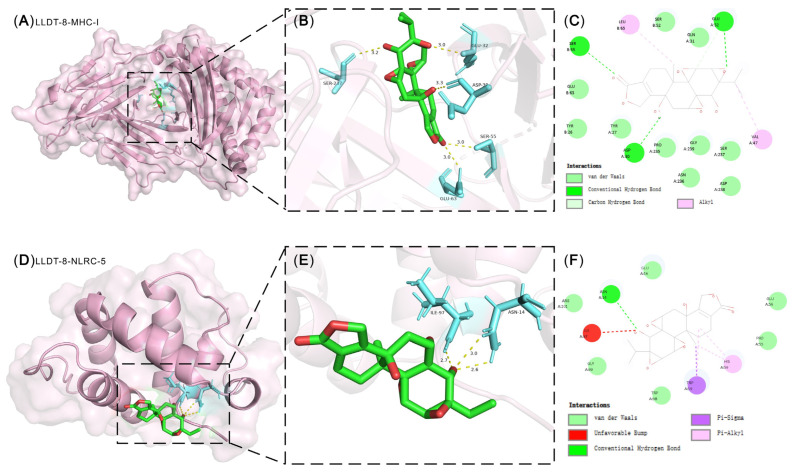
Molecular docking analysis of LLDT-8 with target proteins. LLDT-8 was predicted to bind within the cavity pocket of MHC-I (**A**) and NLRC5 (**D**). (**B**,**E**) illustrate hydrogen bond interactions between LLDT-8 and key amino acid residues, with yellow dashed lines indicating hydrogen bonds. (**C**,**F**) show the two-dimensional interaction diagrams of LLDT-8 with MHC-I and NLRC5, respectively; green dashed lines represent hydrogen bonds, and pink dashed lines indicate π-alkyl interactions. Pi-pi interaction is depicted with pink dotted lines. Red dashed lines denote an unfavorable bump (There may be atomic overlaps that cause van der Waals repulsion). The purple dotted line represents the Pi-Sigma interaction.

Molecular docking analysis was performed to predict the potential binding affinity between LLDT-8 and its target proteins [36]. Based on the observed regulatory effects of LLDT-8 on NLRC5 and MHC-I expression, we evaluated the binding interactions between LLDT-8 and these proteins using in silico docking approaches (Figure 7), and the docking results suggest that LLDT-8 could stably interact with both NLRC5 and MHC-I, supporting the experimental findings. An absolute docking score greater than 6.3 kcal/mol indicates favorable binding between LLDT-8 and NLRC5, whereas a value exceeding 9.6 kcal/mol suggests strong binding activity between LLDT-8 and MHC-I (Table 1). These results provide structural evidence that LLDT-8 may directly interact with components of the NLRC5/MHC-I axis.

## 3. Discussion

IIMs are characterized by immune-mediated muscle injury, with common clinical manifestations including progressive muscle weakness and fatigue [1,2]. Despite advances in clinical management, the substantial mortality and disability associated with IIM continue to hinder therapeutic development.

Considering the higher prevalence observed in females [3], we followed established practices in female mice in our experimental design according to the previous literature [37,38] and systematically evaluated the pharmacological effects and underlying mechanisms of LLDT-8 in EAM in this study. Our findings demonstrate that LLDT-8 significantly improved clinicopathological manifestations, including muscle weakness, pain sensitivity, and abnormal serum biochemical parameters, while reducing inflammatory infiltration in muscle tissue. Mechanistically, LLDT-8 suppressed the transcriptional regulator NLRC5 and its downstream antigen presentation machinery, thereby mitigating muscle injury in EAM.

Animal models of EAM, which recapitulate the phenotypic and histopathological features of IIM, are essential for overcoming the limitations inherent in human studies [29,37]. In this study, EAM mice exhibited marked weight loss, decreased activity, a reduced pain threshold, and evident myasthenia, consistent with previous reports [37,39], supporting the suitability of this model for mechanistic and therapeutic investigation. Current research suggests that the pathological mechanism of polymyositis is an autoimmune disease mediated by T-cell immunity [16]. An immune response is initiated to counteract the lesion once the body is in a certain pathological state. Peripheral immune organs and tissues, including the spleen and lymph nodes, are the sites where lymphocytes settle and exert immune responses. Our results show that EAM mice displayed reduced proportions of CD3^+^ T cells in the spleen and PBMCs, while significant infiltration of CD4^+^ and CD8^+^ T cells was observed in muscle tissue, which may reflect their migration from peripheral compartments to inflamed muscle tissue. We analyzed the murine muscle tissues through H&E and immunohistochemistry and found that the muscle tissues were accompanied by infiltration of inflammatory cells, including CD8^+^ T cells and CD4^+^ T cells.

Histological examination revealed muscle fiber degeneration and necrosis accompanied by increased expression of NLRC5 and MHC-I. This is an important finding because, as mentioned above, elevated MHC-I expression enhances peptide presentation to T cells, thereby triggering inflammatory responses [18]. Considering the critical role of the NLRC5/MHC-I pathway in the pathogenesis of IIM [21,22], we further examined the expression of its downstream components during the myositis process. Consistent with previous findings [40], we observed significant upregulation of key antigen-processing genes, including *Psmb8*, *Psmb9*, *Tap1*, *Tap2*, and *β2M*, both in vivo and in IFN-γ-treated C2C12 myoblasts and myotubes in vitro. These results reinforce the notion that activation of the NLRC5/MHC-I axis contributes to the amplification of immune responses in inflammatory muscle injury.

Over the past decades, extensive efforts have focused on identifying active components of *Tripterygium wilfordii* Hook F, including triptolide derivatives with improved efficacy and reduced toxicity [24,41]. One such triptolide derivative, LLDT-8 has demonstrated immunomodulatory effects in various autoimmune and inflammatory disorders [24,28]. Moreover, our previous work suggested that LLDT-8 may inhibit IFN-γ signaling and reduce interferon-inducible T-cell chemoattractants [42]. In the present study, LLDT-8 suppressed the aberrant expression of NLRC5 and MHC-I-related antigen-processing molecules, thereby potentially attenuating immune activation and inflammatory cell recruitment to injured muscle. Consistent with this mechanism, LLDT-8 significantly reduced CD4^+^ and CD8^+^ T-cell infiltration in muscle tissue, while exerting only modest effects on T-cell proportions in the spleen and PBMCs of EAM mice. These findings suggest that LLDT-8 primarily modulates local inflammatory responses rather than inducing systemic T-cell depletion. Although the precise mechanism by which LLDT-8 regulates NLRC5 expression still needs to be elucidated, it may be associated with its general transcriptional inhibitory properties [25]. Furthermore, in vitro experiments in IFN-γ-stimulated C2C12 myoblasts and myotubes confirmed that LLDT-8 suppresses activation of the NLRC5/MHC-I pathway, reducing both NLRC5 and MHC-I expression as well as downstream antigen-processing molecules, including Psmb8, Psmb9, Tap1, Tap2, and β2M.

## 4. Materials and Methods

### 4.1. Materials

LLDT-8 (purity ≥ 98.5%) was provided by Shanghai Pharmaceuticals Holding Co., Ltd. (Shanghai, China) and dissolved in 0.2% hydroxypropyl methylcellulose (Sigma-Aldrich, St. Louis, MO, USA) prior to use. MP (TargetMol, Boston, MA, USA) was dissolved in sterile distilled water and used as a positive control drug.

### 4.2. Animals

Forty female BALB/c mice (6–8 weeks old; 18–22 g) were purchased from Beijing Huafukang Biological Technology Co., Ltd. (Beijing, China). Two female guinea pigs (10 weeks old; 350–400 g) were obtained from Shanghai Jiesijie Experimental Animal Co., Ltd. (Shanghai, China). All animals were housed under specific pathogen-free conditions at the animal facility of the Shanghai Institute of Materia Medica (Shanghai, China). The environmental conditions were maintained at 22 ± 3 °C with 50 ± 5% relative humidity and a 12 h light/dark cycle. Animals were allowed free access to food and water throughout the study. The animal study was approved by the Institutional Animal Care and Use Committee at Shanghai Institute of Materia Medica (2019-12-ZJP-113) and conducted in accordance with the National Institutes of Health Guide for Care and Use of Laboratory Animals.

### 4.3. Murine EAM Model and Drug Administration

Mice were randomly assigned to five groups (*n* = 8 per group) based on baseline muscle strength and body weight: normal, vehicle, MP, LLDT-8 (0.0625 mg/kg), and LLDT-8 (0.125 mg/kg). Mice in all groups except the normal group were subjected to induction of EAM with slight modifications as previously described [37]. Immunization was performed twice at a one-week interval. Briefly, 1.5 mg of myosin was emulsified thoroughly with an equal volume of complete Freund’s adjuvant (Sigma-Aldrich) containing Mycobacterium tuberculosis H37Ra at a final concentration of 5 mg/mL. A total volume of 200 µL of the emulsion was administered intramuscularly into the right hind limb for the first immunization and subcutaneously at the base of the tail for the second immunization. Pertussis toxin (PT; List Biological Laboratories, Campbell, CA, USA) was administered intraperitoneally at the time of immunization [29,38]. The vehicle group received oral administration of 0.2% hydroxypropyl methylcellulose. MP was applied as a positive control at an oral dose of 10 mg/kg. The LLDT-8 groups were administered LLDT-8 at doses of 0.0625 mg/kg or 0.125 mg/kg. All the mice were administered orally daily from the first immunization until the end of the experiment (Figure 1A).

### 4.4. Evaluation of Murine Muscle Strength

Muscle strength was evaluated by assessing both muscular endurance and grip strength. Muscular endurance was measured using a modified inverted screen test as previously described [29]. Briefly, each mouse was placed on a wire mesh screen, which was then gently inverted by the operator. The time until the mouse fell from the screen was recorded, and the average drop time was used as the endurance value. Grip strength was measured using a digital push–pull force gauge (Model SF-2-500; Aipli, Hangzhou, China) that was reset to zero before testing. Each mouse was gently positioned to grasp the grid of the device with all four limbs and was then steadily pulled backward by the tail until it released the grid, and the peak force was recorded as the grip strength value. Each measurement was repeated three times by the same operator, and the mean value was calculated. Mice were acclimated to the endurance test for one week prior to formal assessment. Muscle strength evaluations were conducted on days 0, 4, 11, and 18 following induction.

### 4.5. Evaluation of Clinical Scores

Body weight was recorded on days 0, 4, 11, and 18 after induction. Clinical severity was assessed based on appearance and behavioral changes according to previously described criteria [29], mice being assigned scores ranging from 0 to 3. Grade 0 indicated no obvious myasthenia; grade 1 represented mild weakness, manifested as reduced vocalization or diminished activity; grade 2 indicated moderate weakness, characterized by a hunched posture, drooping head, flexed forelimbs, and tremors during ambulation or at rest; grade 3 represented severe myasthenia, including absence of vocalization, marked weight loss, muscle atrophy, and labored breathing approaching a moribund state. Each measurement was conducted by the same operator, and the mean value was calculated.

### 4.6. Hot Plate Test

Thermal nociceptive sensitivity was assessed using a hot plate apparatus (Model YSL-6B; Yuyan Scientific Instrument Co., Ltd., Jinan, China). The device was preheated to the preset temperature prior to testing, then each mouse was gently placed on the heated surface, and the latency to the first paw-licking response was recorded as the pain threshold. To ensure reliability, three consecutive trials were performed by the same operator for each mouse with appropriate intervals between tests, and the mean latency was calculated as the final pain threshold value.

### 4.7. Blood Biochemical Analysis

At the experimental endpoint, orbital blood samples were collected under anesthesia and allowed to clot at 4 °C for 4 h. Serum was separated by centrifugation at 12,000 rpm for 10 min and was measured using an automatic biochemical analyzer (E170; Hitachi High-Technologies Corporation, Tokyo, Japan) according to the manufacturer’s instructions. Serum biochemical parameters include LDH (Shanghai Zhicheng Biotechnology Co., Ltd., Shanghai, China), AST (Nanjing Jiancheng Bioengineering Institute, Nanjing, China), and ALT (Nanjing Jiancheng Bioengineering Institute).

### 4.8. H&E and Immunohistochemical Staining

The proximal quadriceps femoris muscle from the right hind limb was harvested and fixed in 4% paraformaldehyde (Servicebio, Wuhan, China), and H&E and immunohistochemical staining were performed as described previously [27]. After routine processing, muscle tissues were embedded in paraffin and sectioned at a thickness of 8–10 µm for H&E staining. Stained sections were scanned and imaged using a NanoZoomer Digital Slide Scanner (Model 2.0-HT; Hamamatsu Photonics K.K., Hamamatsu, Japan), and, images were analyzed with NDP.view2 software (Hamamatsu). The degree of inflammatory infiltration was qualitatively graded into four categories as previously described [29].

For immunohistochemical analysis, paraffin-embedded sections were deparaffinized and rehydrated, and then antigen retrieval was performed. Endogenous peroxidase activity was quenched by incubation with 3% hydrogen peroxide in phosphate-buffered saline for 20 min. After blocking with 5% bovine serum albumin, sections were incubated overnight at 4 °C with rat anti-mouse MHC class I antibody (1:200; Novus Biologicals, Centennial, CO, USA) or rabbit anti-CD8α polyclonal antibody (1:700; Servicebio). After washing, appropriate secondary antibodies (Servicebio) were applied for 10 min, and visualization was performed with 3,3′-diaminobenzidine and counterstaining with hematoxylin. Slides were scanned using the NanoZoomer Digital Slide Scanner (S60; Hamamatsu). Immunohistochemical scores were determined based on the number and proportion of positively stained muscle fibers.

### 4.9. Flow Cytometry Analysis

Spleens and peripheral blood were collected from mice to prepare single-cell suspensions. Red blood cells were lysed where necessary prior to staining. Cells were first incubated with a viability dye for live/dead discrimination, and then blocked with anti-CD16/CD32 monoclonal antibody (BD Biosciences, Franklin Lakes, NJ, USA) to prevent nonspecific Fc receptor binding. Cells were then stained with fluorochrome-conjugated antibodies (BD Biosciences), including FITC-conjugated anti-CD4, BV421-conjugated anti-CD3e, and PerCP-Cy5.5-conjugated anti-CD8a, according to the manufacturer’s instructions. After washing, lymphocyte subsets were analyzed using a BD LSRFortessa flow cytometer (BD Biosciences), and data were processed and analyzed using FlowJo software (Version X; Tree Star Inc., Ashland, OR, USA).

### 4.10. Cell Culture and Treatment

The murine myoblast cell line C2C12 (National Collection of Authenticated Cell Cultures, Shanghai, China) was kindly provided by Professor Lengyin. Cells were cultured in Dulbecco’s modified Eagle’s medium (4.5 g/L glucose; Thermo Fisher Scientific Inc., Waltham, MA, USA) supplemented with 10% fetal bovine serum (Cytiva Bio-technology (Hangzhou) Co., Ltd., Hangzhou, China), 100 U/mL penicillin, and 100 μg/mL streptomycin. Cells were maintained at 37 °C in a humidified incubator with 5% CO_2_.

To induce myogenic differentiation, C2C12 myoblasts were seeded in culture dishes (Corning Inc., Corning, NY, USA) and allowed to reach approximately 85% confluence. The growth medium was then replaced with differentiation medium containing 2% horse serum (Thermo Fisher Scientific Inc.), and cells were cultured for 4–5 days until multinucleated myotubes formed.

For inflammatory stimulation, C2C12 myoblasts or differentiated myotubes were treated with IFN-γ (BD Biosciences) for the indicated durations. For the pharmacological intervention, cells were treated with LLDT-8 at the specified concentrations and time points according to the experimental design.

### 4.11. Cell Viability Assay

Cell viability was evaluated using the MTT assay as previously described [43]. C2C12 cells were seeded into 96-well plates and allowed to adhere. Myoblasts or differentiated myotubes were then treated with graded concentrations of LLDT-8 (25, 50, 100, and 200 nM) for 44 h. Subsequently, MTT reagent (Sigma-Aldrich) was added to each well and incubated for 4 h at 37 °C. After removal of the culture medium, 200 μL of dimethyl sulfoxide (Sigma-Aldrich) was added to dissolve the formazan crystals. Absorbance was measured at 490 nm using a microplate reader (Model 650; Bio-Rad Laboratories, Hercules, CA, USA). Cell viability was demonstrated as a percentage relative to the untreated control group.

### 4.12. Immunofluorescence Staining

C2C12 myoblasts were seeded in 24-well plates for immunofluorescence analysis. Myoblasts and differentiated myotubes were treated with IFN-γ (60 ng/mL) in the presence or absence of LLDT-8 for 48 h. After treatment, cells were washed three times with phosphate-buffered saline, fixed in 4% paraformaldehyde for 20 min, and permeabilized with 1% Triton X-100 for 10 min. Following additional washes, cells were blocked with an immunostaining blocking reagent (Beyotime Biotechnology Co., Ltd., Shanghai, China) at room temperature for 1 h. Cells were then incubated overnight at 4 °C with purified anti-mouse H-2K^k antibody (1:200; BioLegend, San Diego, CA, USA). After washing, cells were incubated with Alexa Fluor^®^ 647-conjugated goat anti-rat IgG secondary antibody (1:200; Abcam Ltd., Cambridge, UK) for 1 h at room temperature in the dark. Nuclei were counterstained with 4′,6-diamidino-2-phenylindole (Beyotime). Fluorescence images were captured using an Olympus inverted fluorescence microscope (Model IX73; Olympus Corporation, Tokyo, Japan).

### 4.13. qRT-PCR

C2C12 myoblasts were seeded in 6-well plates for subsequent experiments. Following treatment with IFN-γ and/or LLDT-8, both cultured cells (myoblasts or differentiated myotubes) and murine muscle tissues were lysed using TRIzol reagent (Thermo Fisher Scientific Inc.). Total RNA was extracted using an RNA simple Total RNA Kit (Tiangen Biotech Co., Ltd., Beijing, China). cDNA was synthesized using the All-in-One cDNA Synthesis SuperMix (Biotool, LLC, Houston, TX, USA). Quantitative PCR was performed using SYBR^®^ Green Real-time PCR Master Mix (Toyobo Co., Ltd., Osaka, Japan) on a 7500 Real-Time PCR System (Applied Biosystems, Foster City, CA, USA). The primer sequences for mouse genes are listed in Table 2. Relative gene expression levels were calculated using the 2^−ΔΔCt^ method, with GAPDH serving as the endogenous reference gene.

### 4.14. Western Blotting

C2C12 cells were seeded in 6-well plates and allowed to adhere overnight before treatment with IFN-γ and/or LLDT-8 for 15 or 30 min, as indicated. Cells were lysed in sodium dodecyl sulfate lysis buffer (Beyotime) supplemented with a protease inhibitor cocktail (F. Hoffmann-La Roche Ltd., Basel, Switzerland). Protein concentrations were determined using a BCA Protein Assay Kit (Thermo Fisher Scientific Inc.). Equal amounts of protein (30 μg per sample) were separated by 8–10% sodium dodecyl sulfate-polyacrylamide gel electrophoresis and transferred onto nitrocellulose membranes. Membranes were blocked with 5% bovine serum albumin and incubated overnight at 4 °C with NLRC5 polyclonal antibody (1:1000; Signalway Antibody LLC, College Park, MD, USA) or anti-MHC-I antibody (1:3000; Novus Biologicals, Centennial, CO, USA). After washing, membranes were incubated with horseradish peroxidase-conjugated anti-rat IgG (1:3000; Cell Signaling Technology, Inc., Danvers, MA, USA) or horseradish peroxidase-conjugated anti-rabbit IgG (1:20,000; Bio-Rad Laboratories) for 1 h at room temperature. Signals were detected with an ECL system. Protein bands were visualized using the SuperSignal™ West Pico PLUS Chemiluminescent Substrate (Thermo Fisher Scientific Inc.) and imaged with a KwikQuant Imager (Kindle Biosciences LLC, Greenwich, CT, USA), and band intensities were quantified using ImageJ software (Version 1.42; National Institutes of Health, Bethesda, MD, USA). Protein expression levels were normalized to GAPDH (KangCheng Biotechnology Co., Ltd., Shanghai, China) and expressed as relative values.

### 4.15. Molecular Docking

Molecular docking analysis was performed as previously described [36]. Briefly, three-dimensional structures of the target proteins were retrieved from the Protein Data Bank. Co-crystallized ligands were removed using PyMOL (Version 3.0.3). The chemical structure of LLDT-8 was obtained from the PubChem database. Water molecules were removed, and hydrogen atoms were added using AutoDock Tools (Version 1.5.7). Polar hydrogen atoms were further assigned, and the docking grid box was defined using AutoDock Tools. Docking simulations were conducted with AutoDock Vina (Version 1.5.7) using default parameters unless otherwise specified. The resulting docking poses were visualized and analyzed using PyMOL (Version 3.0.3) and Discovery Studio 2025 to evaluate binding conformations and intermolecular interactions.

### 4.16. Statistical Analysis

Data are presented as Mean±SD or SEM, as indicated in the figure legends. Statistical analyses were performed using one-way or two-way analysis of variance followed by Dunnett’s multiple comparisons test. All analyses were conducted using GraphPad Prism 8.0 (GraphPad Software, San Diego, CA, USA). A two-tailed *p* value < 0.05 was considered statistically significant.

## 5. Conclusions

This study demonstrates that LLDT-8 alleviates inflammatory muscle injury in EAM by targeting the NLRC5/MHC-I pathway, thereby providing new mechanistic insight and potential therapeutic implications for IIM. In our experiments, LLDT-8 effectively attenuated immune-mediated muscle damage by suppressing aberrant activation of antigen presentation machinery. However, several limitations should be acknowledged. First, although NLRC5 overexpression was closely associated with disease severity, this study did not directly establish NLRC5 as the initiating driver of muscle injury. Second, although the molecular docking analysis supports the strong binding affinity of LLDT-8 to NLRC5 and MHC-I, additional experimental validation (e.g., surface plasmon resonance or co-immunoprecipitation) is needed. Third, the exclusive use of female BALB/c mice is also a limitation. Finally, the precise molecular mechanisms by which LLDT-8 regulates NLRC5 and MHC-I expression still need to be fully elucidated. Further investigations are warranted to clarify these regulatory pathways and to validate the therapeutic potential of LLDT-8 in translational and clinical settings.

## Figures and Tables

**Table 1:** Molecular docking results of LLDT-8 with two target proteins.

Component	Binding Affinity (kcal·mol^−1^)	
	NLRC5	MHC-I
LLDT-8	−6.3	−9.6

**Table 2:** Primer sequenced for qRT-PCR.

Gene	Forward	Reverse
*H2-K1*(*MHC-I*)	CAGGTGGAGCCCGAGTATTG	CGTACATCCGTTGGAACGTG
*β*_2_ *M*	TTCTGGTGCTTGTCTCACTGA	CAGTATGTTCGGCTTCCCATTC
*NLRC5*	GTGCCAAACGTCCTTTTCAGA	AGTGAGGAGTAAGCCATGCTC
*Tap1*	GGACTTGCCTTGTTCCGAGAG	GCTGCCACATAACTGATAGCGA
*Tap2*	CTGGCGGACATGGCTTTACTT	CTCCCACTTTTAGCAGTCCCC
*Psmb8*	ATGGCGTTACTGGATCTGTGC	CGCGGAGAAACTGTAGTGTCC
*Psmb9*	CATGAACCGAGATGGCTCTAGT	GCAAACGAGACATCATAGGCA
*GAPDH*	GGCTCCTTCTGTCGAGTGAC	CGCGGAGAAACTGTAGTGTCC

## Data Availability

The original contributions presented in this study are included in the article. Further inquiries can be directed to the corresponding authors.

## Abbreviations

IIMs	Idiopathic inflammatory myopathies
LLDT-8	(5R)-5-hydroxytriptolide
EAM	Experimental autoimmune myositis
NLRC5	Nucleotide-binding oligomerization domain receptor caspase recruitment domain 5
MHC-I	Major histocompatibility complex-I
Psmb	Proteasome 20S subunit beta
β2M	Beta-2 microglobulin
Tap	Transporter associated with antigen processing
IFN-γ	Interferon-γ
LDH	Lactate dehydrogenase
AST	Aspartate aminotransferase
ALT	Alanine aminotransferase
MP	Methylprednisolone
HPMC	Hydroxypropyl methylcellulose
PT	Pertussis toxin
H&E	Hematoxylin and Eosin
PBMC	Peripheral blood mononuclear cell
